# COVID-19 Infection and Acute Pancreas Transplant Graft Thrombosis

**DOI:** 10.7759/cureus.34087

**Published:** 2023-01-23

**Authors:** Umasankar Mathuram Thiyagarajan, Khaled Dajani, Blaire Anderson, David Bigam, A M James Shapiro

**Affiliations:** 1 Department of Hepatobiliary and Pancreatic Surgery and Transplantation, University of Alberta Hospital, Edmonton, CAN; 2 Department of Surgery, Medicine, and Surgical Oncology, University of Alberta Hospital, Edmonton, CAN

**Keywords:** covid-19, surgical acute abdomen, acute abdomen in covid-19, transplant pancreatectomy, graft failure, pancreas transplantation, graft thrombosis

## Abstract

The coronavirus disease 2019 (COVID-19) pandemic created an unprecedented challenge for healthcare, and the world continues to struggle in recovering from its aftermath. COVID-19 has been clearly linked to hypercoagulable states and can lead to end-organ ischemia, morbidity, and mortality. Immunosuppressed solid organ transplant recipients represent a highly vulnerable population for the increased risk of complications and mortality. Early venous or arterial thrombosis with acute graft loss after whole pancreas transplantation is well-described, but late thrombosis is rare. We herein report a case of acute, late pancreas graft thrombosis at 13 years post pancreas-after-kidney (PAK) transplantation coinciding with an acute COVID-19 infection in a previously double-vaccinated recipient.

## Introduction

Coronavirus disease 2019 (COVID-19) is an infectious disease caused by the severe acute respiratory syndrome coronavirus 2 (SARS-CoV-2) virus, which affected 657 million people and caused 6.6 million deaths worldwide [1]. The impact of COVID-19 on healthcare systems has been immense and unprecedented [2,3]. Acute COVID-19 infection has been associated with a prothrombotic state that in some cases may culminate in end-organ damage from intravascular thrombosis [4]. The activation of vascular endothelial cells, the formation of antiphospholipid antibodies, the proinflammatory cytokine-related aberration of homeostatic systems, and hypercoagulability likely all play a part in causing these thrombotic events in COVID-19 [4,5].

There have been reports of COVID-19-related thrombotic acute graft loss in renal transplant recipients since the pandemic [6,7]. Moreover, there was also a pancreas graft loss in COVID-19 infection that has been reported once previously to our knowledge [8].

## Case presentation

A 48-year-old male with a 41-year history of type 1 diabetes developed an end-stage renal disease in 2003 and underwent a living-unrelated kidney transplantation in 2004. The kidney transplantation technique was standard renal artery and vein to the recipient’s right common iliac artery and vein.

He also suffered from hypoglycemic unawareness and was chronically immunosuppressed and underwent a pancreas-after-kidney (PAK) transplantation in 2008 from a neurologically deceased donor. As the renal graft was anastomosed to the right iliac vessels, the pancreatic blood supply (inflow) was derived from the left common iliac artery (donor iliac artery with internal and external branches anastomosed to the superior mesenteric artery and splenic artery, respectively, in a standard “Y-graft” configuration) with portal venous drainage to the recipient superior mesenteric vein. The donor duodenal loop was anastomosed side to side to the recipient jejunum. Perioperative immunosuppression consisted of basiliximab induction (Simulect, Novartis Pharmaceuticals Canada Inc., Quebec, Canada) intraoperatively and repeated on day 4, tacrolimus, and mycophenolate mofetil, with corticosteroid taper.

He underwent a re-laparotomy and washout for a peripancreatic collection in the second postoperative week. Otherwise, he had an uneventful postoperative course and the following years with normal cross-sectional imaging showing patent vessels. His past medical history also includes hypertension, depression, and chronic hip pain; his medications included aspirin, metoprolol, ramipril, acetaminophen, and vitamin D3. Maintenance immunosuppression consisted of tacrolimus, azathioprine (exchanged due to the intolerance of mycophenolate mofetil), and prednisone with no rejection episodes. As he was an immunosuppressed transplant recipient, he received two doses of COVID-19 vaccinations (messenger RNA {mRNA}-based vaccine), and his antibody-level response was satisfactory.

In 2022, 13 years post pancreas transplant, he developed COVID-19 infection of two-week duration, initially presenting with mild fever, myalgia, and poor appetite but improving in the second week. He then developed vague abdominal pain, mild initially, centrally located, and dull in nature. This progressed over the next 12 hours and was associated with nausea leading to attendance in the emergency room (ER).

At hospital presentation, he was afebrile, and his blood pressure was 143/92 mm Hg, pulse 85/minute, respiratory rate 16/minute, and oxygen saturation 99% on room air. He was 1.72 m in height and 66.8 kg in body weight, with a body mass index of 22.6. The abdominal examination revealed diffuse tenderness with a rebound noted. Urinalysis was negative, COVID-19 polymerase chain reaction (PCR) and rapid COVID-19 tests from the nose/throat remained positive and had been noted to be so on rapid testing seven days prior. The blood investigations showed some abnormal results (Table 1); they were within the normal range previously except for renal functions with chronic allograft nephropathy. The chest X-ray showed only mild atelectasis at the lung bases and no signs of pulmonary COVID-19 involvement.

**Table 1:** Clinical laboratory reference values

Parameters	Patient’s values	Reference values
Hemoglobin	141 g/L	115-155 g/L
White cell count (WCC)	10.5 × 10^9^/L	3.5-10.5 × 10^9^/L
Platelets	247 × 10^9^/L	130-380 × 10^9^/L
Bilirubin	47 µmol/L	3-17 µmol/L
Alanine aminotransferase (ALT)	18 IU/L	17-63 IU/L
Aspartate aminotransferase (AST)	43 IU/L	15-34 IU/L
Alkaline phosphatase (ALP)	112 IU/L	40-120 IU/L
Lipase	109 U/L	0-60 IU/L
Amylase	510 U/L	25-115 IU/L
Glucose	5.9 mmol/L	4.0-11.0 mmol/L
D-dimers	1.44 mg/L	<0.5 mg/L
Lactate	0.8 mmol/L	0.5-2.5 mmol/L
Ferritin	84 µg/L	24-336 µg/L
Urea	13.5 mmol/L	2.1-8.0 mmol/L
Creatinine	195 µmol/L	49-93 µmol/L
International normalized ratio (INR)	1.2	0.9-1.2

**Figure 1:**
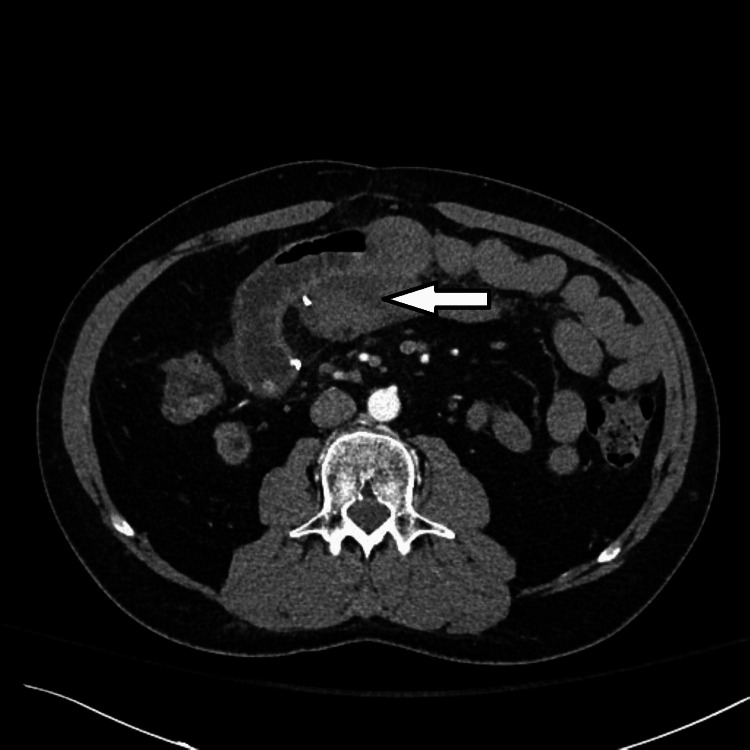
Transverse section of contrast-enhanced abdominal CT scan showing hypoenhancing pancreatic graft CT: computed tomography

**Figure 2:**
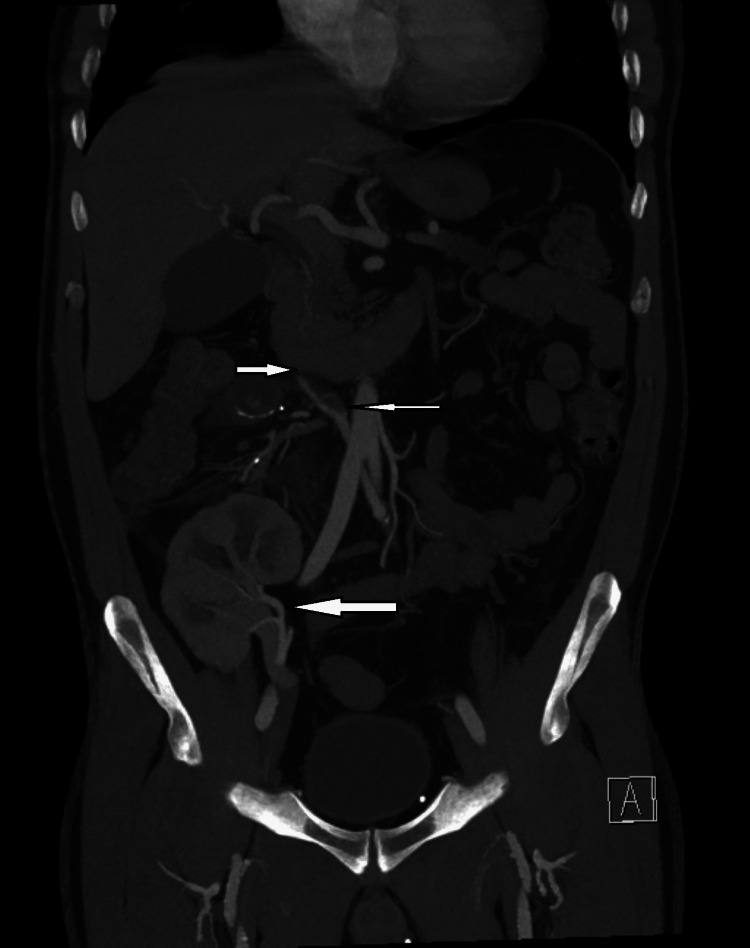
Coronal section of abdominal CT scan showing a thrombosed vascular graft (upper and middle small white arrows) and patent renal graft (lower large single white arrow) CT: computed tomography

He underwent an urgent noncontract computed tomography (CT) scan with contrast avoided due to elevated urea and creatinine. However, due to his COVID-19 illness and concerning peritoneal signs on abdominal examination, a triphasic-phase contrast-enhanced abdominal CT was then obtained, which revealed complete thrombosis of the splenic Y-graft and near-complete thrombosis of the superior mesenteric artery Y-graft to the pancreas (Figures 1-2). The scan also revealed a peri-graft fluid collection, poor enhancement of the body and tail, and marked graft pancreatitis. Fortunately, the transplanted kidney was found to be normal with no vascular compromise at the right iliac fossa (Figure 2). The pancreas transplant was not salvageable by nonoperative means, and surgical exploration proceeded. At laparotomy, most of the pancreas graft was infarcted as was the duodenal loop, with absent flow in the Y-graft, and thus, a transplant pancreatectomy was completed with the ligation of the proximal iliac arterial conduit and the reanastomosis of the small bowel by side-to-side technique. The pathological examination confirmed a pancreatic tissue with multifocal liquefactive necrosis and aggregates of fibrin and neutrophils, compatible with thrombotic infarction and fat necrosis.

He made an uneventful recovery following surgery, and he is being followed by the transplant nephrology and diabetes team. He is also being considered for an islet-after-pancreas transplant and is not deemed suitable for pancreas retransplantation.

## Discussion

It is well-recognized that active COVID-19 infection is associated with a hypercoagulable state of multifactorial cause [4]; this is of principal concern in solid organ transplant recipients where arterial thrombosis or derangements in homeostasis lead to graft loss [6-8]. There has been advancements in practice for thromboprophylaxis in acute COVID-19 [9-11]; postvaccination COVID-19 illness tends to be milder in nature [12-14] and with no reports to date of vascular thrombosis post transplant. Herein, we describe a cautionary case report of acute pancreas graft thrombosis at 13 years post transplant, temporarily associated with acute COVID-19 illness in a double-vaccinated transplant recipient. Low-dose aspirin prophylaxis (81 mg per day) was insufficient in this case to prevent this complication.

High D-dimer and ferritin levels were shown in association with severe illness, morbidity, and mortality in COVID-19 [15,16]. D-dimer is a fibrin degradation product generated by activating fibrinolysis on the blood clot and useful in diagnosing venous thromboembolism [17]. As expected, an initial case report of renal graft thrombosis was noted to have high D-dimer levels and ferritin levels [7]. A second case report of renal graft thrombosis was noted in a patient who underwent a simultaneous pancreas and kidney (SPK) transplant. The biochemical profile again showed high levels of D-dimer/ferritin; however, the patient was known to have transplant renal artery stenosis, treated with angioplasty prior to the COVID-19 illness, and therefore was at risk of precipitous thrombosis [6].

A similar case of possible COVID-19-related pancreas transplant graft thrombosis was reported in 2022 [8]. The authors identified obesity as the dominant risk factor for graft thrombosis in COVID-19, but our patient was of normal body habitus. Moreover, the prior report of graft thrombosis occurred at four months after COVID-19, which seems of questionable association. This patient also did not have an estimation of D-dimer and ferritin levels.

Our patient developed acute graft thrombosis in the second week of the COVID-19 illness, with only mild elevation in D-dimer and normal ferritin, lactate, and glucose levels. Presumably, minimal arterial remnant flow into the pancreas graft preserved endocrine islet function that masked acute hyperglycemia typically associated with pancreas transplant graft thrombosis. A high index of suspicion in active COVID-19 infection should prompt the early use of contrast-enhanced CT imaging, despite acute kidney injury. The early diagnosis of this irreversible state and prompt surgical management will minimize perioperative risk and accelerate recovery.

The degree and duration of the COVID-19 prothrombotic state in transplant recipients remain unknown. Among the four cases, except one, thrombosis developed within four weeks of COVID-19 infection, which may serve as a guide to the duration of extended thromboprophylaxis in this setting. From our own anecdotal experience, low-dose aspirin alone is insufficient to prevent this complication.

The limitations of our study include the temporal association with acute COVID-19 infection, but it is not absolute proof that this was causative. We also do not have access to contrast CT imaging shortly prior to the acute thrombosis to demonstrate that the Y-graft and transplant pancreatic arterial inflow was completely normal preceding the infection. A preexisting stenosis could perhaps have led to a thrombotic tendency, although this was not evident on prior imaging.

## Conclusions

A high index of suspicion and prompt arterial-phase contrast-enhanced abdominal cross-sectional imaging is important for prompt diagnosis and management of acute pancreas transplant graft thrombosis, especially in the setting of acute or recent COVID-19 infection. The occurrence of thrombosis may vary from days to weeks following the COVID-19 illness, and thromboprophylaxis with therapies more potent than aspirin monotherapy may be indicated.
